# Adverse In-Hospital Outcomes after Radical Prostatectomy in Leukemia History Patients

**DOI:** 10.3390/cancers16152764

**Published:** 2024-08-05

**Authors:** Fabian Falkenbach, Francesco Di Bello, Natali Rodriguez Peñaranda, Mattia Longoni, Andrea Marmiroli, Quynh Chi Le, Zhe Tian, Jordan A. Goyal, Nicola Longo, Salvatore Micali, Alberto Briganti, Ottavio de Cobelli, Felix K. H. Chun, Fred Saad, Shahrokh F. Shariat, Lars Budäus, Markus Graefen, Pierre I. Karakiewicz

**Affiliations:** 1 Cancer Prognostics and Health Outcomes Unit, Division of Urology, University of Montreal Health Center, Montreal, QC H2X 3E4, Canada; 2 Martini-Klinik Prostate Cancer Center, University Medical Center Hamburg-Eppendorf, 20251 Hamburg, Germany; 3 Department of Neurosciences, Science of Reproduction and Odontostomatology, University of Naples Federico II, 80138 Naples, Italy; 4 Department of Urology, Ospedale Policlinico e Nuovo Ospedale Civile S. Agostino Estense Modena, University of Modena and Reggio Emilia, 41121 Modena, Italy; 5 Division of Experimental Oncology, Unit of Urology, URI, Urological Research Institute, IRCCS San Raffaele Scientific Institute, 20132 Milan, Italy; 6 Vita-Salute San Raffaele University, 20132 Milan, Italy; 7 Department of Urology, IEO European Institute of Oncology, IRCCS, 20141 Milan, Italy; 8 Università degli Studi di Milano, 20122 Milan, Italy; 9 Department of Urology, Goethe University Frankfurt, University Hospital, 60596 Frankfurt am Main, Germany; 10 Department of Oncology and Haemato-Oncology, Università degli Studi di Milano, 20122 Milan, Italy; 11 Department of Urology, Comprehensive Cancer Center, Medical University of Vienna, 1090 Vienna, Austria; 12 Department of Urology, Weill Cornell Medical College, New York, NY 10065, USA; 13 Department of Urology, University of Texas Southwestern Medical Center, Dallas, TX 75390, USA; 14 Hourani Center for Applied Scientific Research, Al-Ahliyya Amman University, Amman 19111, Jordan; 15 Department of Urology, University Medical Center Hamburg-Eppendorf, 20251 Hamburg, Germany

**Keywords:** NIS, prostatectomy, prostate cancer, leukemia, CLL, pelvic lymph node dissection

## Abstract

**Simple Summary:**

Leukemia history affects some radical prostatectomy (RP) patients. While its prevalence and impact as an adverse risk factor are well-known in cardiac surgery, the number of RP patients with a leukemia history and their rate of adverse in-hospital outcomes are unknown. We identified RP patients with a leukemia history in a large-scale database. Patients with a leukemia history had higher rates of acute kidney injury, more frequent dialysis, and extended hospital stays compared to those without a leukemia history.

**Abstract:**

Introduction: Leukemia history affects some radical prostatectomy (RP) patients. Although its prevalence and effect as an adverse risk factor are well known in cardiac surgery, the number of RP patients with a leukemia history, as well as their rate of adverse in-hospital outcomes, are unknown. Methods: We identified RP patients (National Inpatient Sample 2000–2019), stratified according to the presence or absence of a leukemia history. Descriptive analyses, propensity score matching (PSM, ratio 1:10), and multivariable logistic regression models were used. Results: Of 259,939 RP patients, 416 (0.2%) had a leukemia history. Their proportion increased from 0.1 to 0.2% covering the study span (*p* < 0.01). Leukemia history patients were older (median age, 64 vs. 62 years, *p* < 0.001). After PSM for age, insurance status, ethnicity, pelvic lymph node dissection, and Charlson Comorbidity Index, leukemia history RP patients exhibited higher rates of acute kidney injury (<2.6 vs. 0.9%; Odds Ratio [OR] 2.0, *p* = 0.02), more frequently underwent dialysis (3.6 vs. 1.9%; OR 1.9, *p* = 0.03), and more frequently had a length of stay exceeding one week (4.8 vs. 2.5%; OR 2.0, *p* = 0.006). Conclusions: Although leukemia history RP patients are rare, their numbers have increased. Renal complications and extended hospital stays are more frequent in those individuals.

## 1. Introduction

Radical prostatectomy (RP) patients occasionally harbor a history of a previous malignancy. Of those, some may represent solid cancers and others may be hematological cancers, of which the most frequent is leukemia. Prostate cancer (PCa) is the second most frequent secondary malignancy in leukemia patients after skin cancer [1,2,3]. However, it is unclear how many RP patients actually exhibit a history of leukemia, although several case reports and case series from renowned investigators like Jonathan I. Epstein, Patrick C. Walsh, and Martha K. Terris previously addressed this topic [4,5,6,7,8,9,10,11]. The majority of these studies focused on pathological findings originating from pelvic lymph node dissection specimens, where evidence of leukemia was identified in the form of lymph node invasion (LNI). Indeed, LNI from leukemia or PCa, as well leukemia history itself, may predispose patients to higher rates of adverse in-hospital outcomes after RP. Indeed, leukemia history patients exhibited less favorable in-hospital outcomes after several other surgical procedures, and especially after cardiac surgery, that included higher rates of complications, such as blood transfusions or renal failure [12,13,14,15,16,17,18,19,20,21,22,23,24,25,26]. Despite the richness of information from other surgery types, the prevalence of leukemia history in RP patients as well as its association to higher rates of adverse in-hospital outcomes are unknown.

We addressed both knowledge gaps using a contemporary, large-scale population-based cohort of patients undergoing RP within the National Inpatient Sample (NIS) from 2000 to 2019.

## 2. Materials and Methods

### 2.1. Data Source

To test for adverse in-hospital outcomes after RP, we relied on discharge data from the NIS (2000–2019). The longitudinal NIS databases are included in the Healthcare Cost and Utilization Project (HCUP) and formed by the Agency for Healthcare Research and Quality (AHRQ) within a Federal-State partnership [27]. The International Classification of Disease (ICD) 9th revision Clinical Modification (ICD-9-CM), ICD 10th revision Clinical Modification (ICD-10-CM), and the ICD 10th revision Procedure Coding System (ICD-10-PCS) were used for coding diseases and procedures.

### 2.2. Study Population

We focused on patients aged ≥ 18 years with a primary diagnosis of PCa (ICD-9-CM codes 185, 233.4, 236.5, and V10.46; ICD-10-CM codes C61, D07.5, D40.0, and Z85.46). Only patients treated with RP were included according to the previously reported methodology [28]. Patients were stratified according to diagnostic codes indicative of leukemia history: lymphoid leukemia (ICD-10-CM code C91; ICD-9-CM code 204), myeloid leukemia (ICD-10-CM code C92; ICD-9-CM code 205), monocytic leukemia (ICD-10-CM code C93; ICD-9-CM code 206), other/unspecified leukemias (ICD-10-CM codes C94 and C95; ICD-9-CM code 207 and 208).

### 2.3. Outcomes of Interest

The study endpoints consisted of adverse in-hospital outcomes, defined as intraoperative and postoperative complications (overall, cardiac, renal, genitourinary, vascular, infections, and bleeding), rates of transfusions, critical care therapy (CCT) use, dialysis, long length of stay (>1 week), and in-hospital mortality, identified by ICD-9 and ICD-10 codes according to previously established methodology [29,30]. Procedures like CCT use and dialysis were not included in the definition of overall complications. The Deyo modification of the Charlson Comorbidity Index (CCI) was used to account for comorbidities [31]. We relied on coding algorithms for defining comorbidities using ICD-9-CM and ICD-10-CM codes by Quan et al. [32]. Covariates consisted of patient characteristics including age at admission (years, continuously coded), insurance status (“Medicaid/Medicare”, “Other”, or “Private”), ethnicity (“Caucasian” vs. “Others”), CCI (0 vs. 1 vs. ≥2), and pelvic lymph node dissection at RP (yes vs. no). In leukemia history patients, two points were subtracted from the CCI to compare additional comorbidities besides their hematological condition. Teaching hospital status (“Teaching” vs. “Non-teaching”) was also assessed.

### 2.4. Statistical Analyses

First, patient characteristics, complications, transfusion rates, CCT use, length of stay, and mortality were tabulated. Medians and interquartile ranges (IQR) were recorded for continuously coded variables. Frequencies and proportions were recorded for categorical variables. Wilcoxon rank sum and Pearson Chi-square tests were applied to test for differences between patients with or without a history of leukemia. Second, the estimated annual percentage changes (EAPC) for the percentage of RP patients with a history of leukemia were calculated using least-squares linear regression. Third, propensity score matching (PSM, ratio 1:10) according to the nearest neighbor was performed for age at admission, insurance status, ethnicity, pelvic lymph node dissection at RP, and CCI between RP patients with history of leukemia and RP patients without history of leukemia to maximally reduce the effect of bias and confounding. Fourth, univariable and multivariable logistic regression models addressing adverse in-hospital outcomes were fitted.

Analyses and reporting followed NIS reporting guidelines [27]. The counts and associated proportions were censored for samples <11. R software environment was used for statistical computing and graphics (R version 4.2.2; R Foundation for Statistical Computing, Vienna, Austria) [33]. All tests were two-sided with a significance level of *p* < 0.05. 

## 3. Results

### 3.1. Characteristics of the Study Population

**Figure 1:**
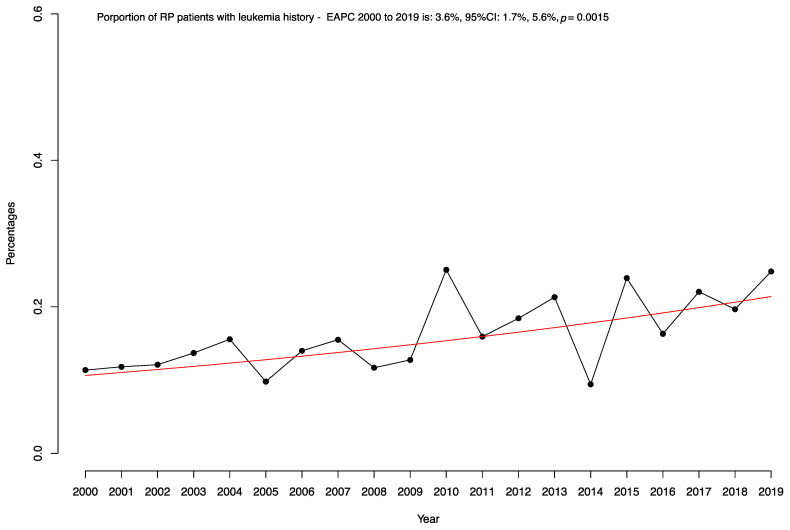
Proportion of prostate cancer patients undergoing radical prostatectomy with the presence of leukemia over time within the National Inpatient Sample (NIS) from 2000 to 2019. The red line represents the estimated annual percentage change from 2000 to 2019.

We identified 259,939 patients who underwent RP between 2000 and 2019. Of these, 416 (0.2%) had a leukemia history (Table 1). From 2000 to 2019, the proportion of patients with a history of leukemia prior to RP increased from 0.1 to 0.2% (EAPC: +3.6%, 95% confidence interval (CI): +1.7 to +5.6%; *p* < 0.01; Figure 1).

Patients with a history of leukemia were older (median 64 vs. 62 years, *p* < 0.001) and more frequently Caucasian (72.6 vs. 63.5%, *p* < 0.001). At RP, leukemia history patients were more frequently treated with concomitant pelvic lymph node dissection (38.7 vs. 33.3%, *p* = 0.02) and more frequently treated at teaching hospitals (71.6 vs. 66.3%, *p* = 0.02). No differences in rates were recorded according to CCI between patients with a history of leukemia and those without.

Relying on 1:10 propensity score matching for age, insurance status, ethnicity, pelvic lymph node dissection, and Charlson Comorbidity Index, of 259,939 RP patients, 416 of 416 (100%) leukemia history patients and 4160 of 259,523 (1.6%) patients without a leukemia history were included in further analyses. After propensity score matching, no statistically significant residual differences remained for all above variables (Table 1).

### 3.2. Adverse In-Hospital Outcomes

Relying on a 1:10 propensity score matched cohort, leukemia history RP patients more frequently had acute kidney injury (<2.6 vs. 0.9%; *p* = 0.006) and more frequently received dialysis (3.6 vs. 1.9%; *p* = 0.04; Table 2). Leukemia history RP patients exhibited higher rates of hospital stays exceeding one week (4.8 vs. 2.5%, *p* = 0.01). After multivariable adjustment, history of leukemia represented an independent predictor for acute kidney injury (odds ratio (OR) 2.0, *p* = 0.02), dialysis (OR 1.9, *p* = 0.03), and hospital stay >1 week (OR 2.0, *p* = 0.006). To further validate these findings, we performed subgroup analyses for patients with acute or subacute leukemia (20/416, 4.8%), leukemia history patients after stem cell or bone marrow transplantation (11/416, 2.6%), and leukemia history patients previously treated with chemotherapy (13/416, 3.1%) or irradiation (<11/416, <2.6%). None of these subgroups exhibited significantly higher rates of acute kidney injury, dialysis, or hospital stay >1 week than those in the overall leukemia history cohort.

Conversely, for the remaining comparison categories (transfusion rates; overall intraoperative, cardiac, genitourinary, vascular, infectious, and bleeding complications; and critical care therapy and mortality), no statistically significant or clinically meaningful differences were recorded (Table 2). Indeed, the overall complication rate in patients with a history of leukemia was 19.7 vs. 17.1% in those without (*p* = 0.2). Neither the rate of critical care therapy <2.6 vs. 0.6%; *p* = 0.7) nor that of in-hospital mortality (0 vs. <0.3%; *p* = 1) was higher in patients with a history of leukemia than in those without.

## 4. Discussion

Leukemia history in RP patients is rare and may predispose them to higher rates of in-hospital complications. Despite a relative abundance of literature addressing leukemia history in other surgeries, specific analyses have never reported the prevalence of leukemia history at RP and its potential association with adverse in-hospital outcomes. We have addressed the two knowledge gaps and made several noteworthy observations.

First, despite the interest in leukemia history at RP [1,4,5,6,7,8,9,10,11], the existing studies cannot provide estimates about the proportion of leukemia history patients at RP and did not address complications, as has been done extensively in regard to cardiac surgery. Indeed, the current English-language medical literature does not offer studies examining leukemia history patients treated by RP, regardless of the endpoint of interest. Such data cannot be provided at a single institution level or even within multi-institutional databases due to their rarity. Only population-based data repositories such as the NIS may provide sufficient numbers of observations. In the current study, relying on data from 2000 to 2019, we recorded 416 leukemia history patients out of 259,939 RP patients (0.2%). This proportion is consistent with the varying incidence rates of leukemia history at RP, ranging from 0.2 to 0.9% in case series [4,8,10]. This estimate is also consistent with the proportions recorded in other surgery types, that is, 0.2% for cardiac surgery patients within a nationally representative cohort of >1.25 million patients in the United States [13].

Second, although RP patients with leukemia history were rare, the proportion of RP patients with leukemia increased in a statistically significant fashion over the course of this study, from 0.1% to 0.2% (*p* = 0.002). Indeed, an increasing rate of leukemia history patients in the context of surgeries other than RP has been described as well [16,17]. This increase validates the pertinence of the current study.

Third, we identified important differences in patient characteristics that distinguished RP patients with a history of leukemia from those without. Specifically, RP patients with a leukemia history were older (median, 64 vs. 62 years), more frequently Caucasian (72.6 vs. 63.5%), more frequently treated with concomitant pelvic lymph node dissection at RP (38.7 vs. 33.3%), and more frequently treated at teaching hospitals (71.6 vs. 66.3%). For instance, Caucasian ethnicity is a known risk factor for leukemia [34], validating this finding. These differences indicated the need for propensity score matching and multi-variable adjustment.

Fourth, of all 13 examined adverse in-hospital outcomes (acute kidney injury, dialysis, and transfusion; overall intraoperative, cardiac, genitourinary, vascular, infectious, and bleeding complications; and critical care therapy, length of stay longer than one week, and mortality), three exhibited significant differences between patients with a history of leukemia and their counterparts without a leukemia history. Specifically, the rate of acute kidney injury was higher in RP patients with a history of leukemia than in their counterparts without (<2.6 vs. 0.9%, *p* = 0.006). In MVA, a history of leukemia predicted a 2-fold higher rate of acute kidney injury (*p* = 0.02). Additionally, higher dialysis rates after RP were observed in patients with a history of leukemia (3.6% vs. 1.9%, *p* = 0.04). In MVA, a history of leukemia predicted a 1.9-fold higher rate of dialysis (*p* = 0.03). Taken together, leukemia history predicted higher rates of kidney-related unfavorable outcomes. This is consistent with findings made in other surgical procedures. Nguyen et al. (n = 37,839) reported significantly higher rates of renal failure (5.4 vs. 2.8%, *p* = 0.03) and dialysis (1.8 vs. 0.3%, *p* = 0.02) in patients with a history of hematologic malignancies compared to their counterparts without [14]. Sommer et al. also reported a tendency towards higher rates of renal failure and dialysis in cardiac surgery patients with a history of hematologic malignancies and prior chemotherapy or irradiation (21.4 vs. 5.3%, *p* = 0.3 or 20.0% vs. 8.7%, *p* = 0.3) [21]. Nonetheless, these associations are not universal, and other studies have failed to exhibit higher rates of kidney-related adverse outcomes after cardiac surgery in leukemia history patients [12,13,23]. As a consequence, further validation studies in leukemia history RP patients in large-scale databases outside the United States are warranted.

Fifth, leukemia history patients had a 2-fold higher risk of a hospital stay longer than one week (4.8 vs. 2.5%; OR 2.0, *p* = 0.006). A longer length of stay was also reported in patients with hematological malignancies undergoing orthopedic surgeries [16,17]. However, as for other positive associations, other investigators did not identify a history of leukemia as a predictor for a longer length of stay [12,13,22].

Sixth, despite a convincing body of evidence indicating higher rates of blood transfusions in surgeries other than RP for leukemia history patients [12,13,14,16,21], such an association was not identified in the current study. Specifically, the rate of blood transfusions was 8.9% for leukemia history patients vs. 6.8% for their matched counterparts without a leukemia history (OR 1.4, *p* = 0.1). For instance, Mardigal et al. and Zhu et al. reported significantly higher rates of blood transfusion (34 vs. 28%, *p* = 0.003 and 87 vs. 65%, *p* = 0.001) after cardiac surgery in patients with a history of leukemia within two independent, large-scale databases of over 1.25 million and 50 thousand patients, respectively [12,13]. The differing findings between RP and cardiac surgery may be explained by the inherent differences in the nature of these surgical procedures. Cardiac surgery typically involves higher rates of bleeding, necessitating more frequent blood transfusions, reflecting the distinct hemostatic challenges presented by this type of surgery. However, it is possible that a higher rate of blood transfusions in leukemia history patients after RP may be identified in other large-scale databases.

Taken together, the prevalence of RP patients with a leukemia history is increasing over time in a statistically significant fashion, and these patients are at higher risk for acute kidney injury, dialysis, and an extended length of stay. The observations made in this study are in accordance with the marginal leukemia history rates in other malignancies and the relatively small magnitude of complications differences reported from other procedures. RP surgeons should be aware of these differences when a RP is contemplated in a patient with a history of leukemia.

While an analysis of a rare combination such as this is only possible using large retrospective databases, the present study has inherent limitations due to its reliance on this approach. First and foremost, the limited sample size of RP patients with a history of leukemia undermines the clinical importance of the current findings. Specifically, the observations from the current study may not be pertinent to occasional RP surgeons who may never encounter patients with a history of leukemia. However, the current observations are important for high-volume users, and even more important for high-volume RP centers, where patients with a history of leukemia will be encountered regularly and at an increasing rate. Second, in the current study, we were able to quantify the proportion of RP patients with a history of leukemia. However, the absence of pathological information originating from pelvic lymph node dissection specimens prevented us from quantifying the rate of leukemia LNI. Ideally, leukemia status is defined by a history of leukemia and leukemia LNI. Third, it may very well be assumed that only the most fit and healthy leukemia history patients are referred to RP and that retrospective data like the NIS is prone to selection bias. However, this limitation applies to all current and previous studies that rely on the NIS or similar databases, such as the Surveillance Epidemiology and End Results database. Finally, the NIS only provides data within the hospital stay of the patient. Therefore, it was not possible to assess further complications after the patient was discharged after RP. Moreover, the individual risk of developing adverse in-hospital outcomes in leukemia history patients may be substantially influenced by prior treatments, procedures, and leukemia/cancer characteristics that were not assessed in our analyses. In fact, the NIS offers only limited information on the cancer/leukemia stage and prior medical history. For instance, within leukemia history patients, we identified only 11/416 (2.6%) patients with a prior history of stem cell or bone marrow transplantation, 13/416 (3.1%) patients with a personal history of antineoplastic chemotherapy, and <11/416 (<2.6%) patients with personal history of irradiation. These small numbers restricted us from meaningful subgroup analyses. Our analyses are most representative of chronic leukemia patients without further risk factors, limiting the generalizability of our findings. 

## 5. Conclusions

Patients with a leukemia history undergoing radical prostatectomy were rare and had higher rates of kidney-related adverse in-hospital outcomes and extended length of stay.

## Figures and Tables

**Table 1:** Descriptive characteristics of prostate cancer patients undergoing radical prostatectomy, stratified according to the presence or absence of leukemia history, prior and after propensity score matching (PSM) 1:10.

		Prior PSM			After PSM 1:10		
**Characteristic**		With Leukemia History n = 416 (0.2%)	Without Leukemia History n = 259,523 (99.8%)	*p*-Value ^1^	With Leukemia History n = 416 (9.1%)	Without Leukemia History n = 4160 (98.9%)	*p*-Value ^1^
**Age at admission,** **median (IQR)**	**in years**	64 (60, 69)	62 (57, 67)	<0.001	64 (60, 69)	64 (60, 69)	1
**Insurance, n (%)**	**Medicaid/** **Medicare**	187 (45.0%)	91,057 (35.1%)	<0.001	187 (45.0%)	1887 (45.4%)	1
	**Other**	16 (3.8%)	11,142 (4.3%)		16 (3.8%)	153 (3.7%)	
	**Private**	213 (51.2%)	15,7328 (60.6%)		213 (51.2%)	2120 (51.0%)	
**Ethnicity, n (%)**	**Caucasian**	302 (72.6%)	164,844 (63.5%)	<0.001	302 (72.6%)	3027 (72.8%)	1
	**Others**	114 (27.4%)	94,683 (36.5%)		114 (27.4%)	1133 (27.2%)	
**PLND at RP, n (%)**		161 (38.7%)	86,294 (33.3%)	0.02	161 (38.7%)	1604 (38.6%)	1
**Charlson Comorbidity Index ^†^, n (%)**				0.9			1
	**0**	297 (71.4%)	187,070 (72.1%)		297 (71.4%)	2939 (70.6%)	
	**1**	79 (19.0%)	47,000 (18.1%)		79 (19.0%)	813 (19.5%)	
	**≥2**	40 (9.6%)	25,457 (9.8%)		40 (9.6%)	408 (9.8%)	
**Teaching hospital, n (%)**		298 (71.6%)	172,015 (66.3%)	0.04	298 (71.6%)	2984 (71.7%)	1

^1^ Wilcoxon rank sum test, Pearson’s Chi-square test. Abbreviations: IQR = Interquartile range, PSM = propensity score matching, RP = radical prostatectomy, PLND = pelvic lymph node dissection. ^†^ In patients with a history of leukemia, 2 points were removed to compare the additional comorbidity besides their hematological condition.

**Table 2:** Adverse in-hospital outcomes (overall complications, intraoperative complications, postoperative complications, and mortality) and length of stay in radical prostatectomy patients after 1:10 propensity score matching, stratified according to the presence or absence of a history of leukemia.

Endpoints	With Leukemia History n = 416 (0.2%)	Without Leukemia History n = 4160 (98.9%)	*p*-Value ^1^	Multivariable OR (95% CI) ^2^	*p*-Value ^2^
**Overall complications, n (%)**	82 (19.7%)	713 (17.1%)	0.2	1.2 (0.9, 1.6)	0.2
**Acute kidney injury, n (%)**	<11 (<2.6%)	36 (0.9%)	0.006	2.0 (1.1, 3.5)	0.02
**Dialysis, n (%)**	15 (3.6%)	81 (1.9%)	0.04	1.9 (1.1, 3.2)	0.03
**Transfusion, n (%)**	37 (8.9%)	282 (6.8%)	0.1	1.4 (0.9, 1.9)	0.1
**Intraoperative complications, n (%)**	15 (3.6%)	166 (4.0%)	0.8	0.9 (0.5, 1.5)	0.7
**Cardiac complications, n (%)**	13 (3.1%)	104 (2.5%)	0.5	1.3 (0.7, 2.3)	0.4
**Genitourinary complications, n (%)**	<11 (<2.6%)	62 (1.5%)	0.4	1.5 (0.7, 3.0)	0.3
**Vascular complications, n (%)**	<11 (<2.6%)	23 (0.6%)	1	0.9 (0.1, 3.0)	0.9
**Infectious complications, n (%)**	0 (0%)	11 (0.3%)	0.6	NA	
**Bleeding complications, n (%)**	<11 (<2.6%)	61 (1.5%)	1.0	0.7 (0.2, 1.6)	0.4
**Critical care therapy, n (%)**	<11 (<2.6%)	25 (0.6%)	0.7	1.2 (0.3, 3.5)	0.8
**Length of stay >1 week, n (%)**	20 (4.8%)	106 (2.5%)	0.01	2.0 (1.2, 3.3)	0.006
**In-hospital mortality, n (%)**	0 (0%)	<11 (<0.3%)	1	NA	

^1^ Pearson’s Chi-square test. ^2^ Multivariable regression models predicting post-operative, in-hospital complications according to the presence or absence of a leukemia history in patients undergoing radical prostatectomy after 1:10 propensity score matching (PSM). Multivariable adjustment was made to age at admission, comorbidities, ethnicity, insurance status, pelvic lymph node dissection, and teaching hospital status. Reference: Patients without a history of leukemia. Abbreviations: IQR = Interquartile range, OR = Odds Ratio, CI = Confidence Interval, NA = not assigned.

## Data Availability

The data presented in this study are available in this article.
